# Daily Physical Activity Among Toddlers: Hip and Wrist Accelerometer Assessments

**DOI:** 10.3390/ijerph16214244

**Published:** 2019-11-01

**Authors:** Soyang Kwon, Kyle Honegger, Maryann Mason

**Affiliations:** Ann & Robert H. Lurie Children’s Hospital of Chicago, Chicago, IL 60611, USA; khonegger@luriechildrens.org (K.H.); mmason@luriechildrens.org (M.M.)

**Keywords:** children, early childhood, ActiGraph, waist, sedentary behavior

## Abstract

Physical activity (PA) habits seem to track over time from as young as early childhood. For children under age 3 years, wearable sensor-measured PA levels have begun to be investigated. The aims of this study were to evaluate the feasibility of using hip- vs. wrist-worn accelerometers, and to report accelerometer-derived PA metrics among toddlers. A convenience sample of 22 toddlers aged 13 to 35 months and their mothers were recruited for this study. ActiGraph wGT3X-BT accelerometers were attached to wrist bands and waist belts. The mothers were asked to affix a wrist band and a waist belt to their participating children during waking hours for four days. They also completed an acceptability survey. Of the 22 toddlers, 19 (86%) had ≥ 3 valid days of hip data, while only 14 (64%) did so for wrist data (*p* = 0.16). In terms of acceptability, 18 mothers (82%) responded that the 4-day hip wear was easy, while only 13 (59%) responded that the 4-day wrist wear was easy (*p* = 0.19). Daily light-intensity PA (LPA) was on average 161 min, and daily moderate- and vigorous-intensity PA (MVPA) was on average 47 min, as determined using published hip accelerometer cut-points. There were no significant differences in LPA or MVPA by age or by sex. In conclusion, this study suggests that hip placement of an ActiGraph accelerometer is more feasible than wrist placement among toddlers.

## 1. Introduction

Despite the recognized health benefits of physical activity (PA) [1], a majority of children do not meet the recommended physical activity (PA) level [2]. Children who are inactive at age 5 years tend to remain inactive throughout childhood and adolescence [3]. Recent longitudinal studies have reported that PA behavior tracks at a moderate level from age 1 to 3 years [4], or age 1 to 5 years [5]. Collectively, these findings suggest that PA behaviors could be established at a very young age and sustained over time. Recognizing the emerging need to assess PA behavior among very young children, studies have begun to investigate wearable sensor-measured PA levels among toddlers aged 1 and 2 years [6,7,8,9,10,11,12,13,14,15,16,17,18].

Accelerometers are widely accepted as the best practice for objectively measuring regular PA among children. In particular, ActiGraph accelerometers (Pensacola, FL, USA) are the most commonly used research-grade accelerometers [19]. The ActiGraph accelerometers are relatively small and light devices (e.g., 4.6 cm × 3.3 cm × 1.5 cm and 19 g for the wGT3X-BT model). Several aspects of the device have been shown to be acceptable, such as its size, weight, and its ability to be worn on a hip and a wrist, by attaching it to a waist belt, a waist clip, or a wrist band, for several days, among preschoolers and older children [20]. However, this same level of feasibility cannot be assumed for toddlers, given that their bodies are significantly smaller and their daily life activity patterns are uniquely different (e.g., naps and frequent clothes/diaper changes) from older children. Only a few studies [6,20] have examined the feasibility of using ActiGraph accelerometers among toddlers. These studies [6,20] reported that the use of a hip-worn accelerometer is feasible among toddlers. A paper by Johansson et al. [15] reported the successful completion (89% of the sample) of a wrist-worn accelerometer assessment among toddlers, although it did not explicitly assess feasibility. The placement of the accelerometer (e.g., hip or wrist) is a key methodological issue that has important implications not only for data processing algorithms, but also for adherence to the monitoring protocol. Although hip- and wrist-worn accelerometers are both feasible for use in older children, wrist placement has shown higher compliance than hip-placement [21], while hip-worn accelerometer data have shown higher accuracy in estimating PA than wrist-worn accelerometer data [22,23]. However, to our knowledge, no studies have yet reported on a comparison of the feasibility between hip and wrist placement among toddlers. The aims of this study were to examine the feasibility of using hip vs. wrist-worn ActiGraph accelerometers and to report accelerometer-derived PA metrics among toddlers.

## 2. Methods

### 2.1. Participants

The eligibility criteria for participants were toddlers aged between 13 and 35 months who can independently walk. A total of 22 mothers with eligible toddlers (11 one-year olds and 11 two-year olds; 10 boys and 12 girls) among Lurie Children’s Hospital of Chicago employees and their acquaintances volunteered for their child participation to this study. A $20 gift card was given to each participant for their time and effort. The mothers of the participating children provided written informed consent. This study was approved by the Lurie Children’s Hospital of Chicago Institutional Review Board.

### 2.2. Data Collection

For PA assessment, ActiGraph wGT3X-BT accelerometers (Pensacola, FL, USA) were used. Accelerometers were attached to ActiGraph woven nylon wrist bands and ActiGraph elastic waist belts. During a study visit, research staff instructed participant mothers on how to affix a wrist band and a waist belt and how to complete an accelerometer wear time log sheet. The mother was asked to affix both the waist belt and the wrist band simultaneously to her participating child during the child’s waking hours for four consecutive days, including at least one weekend day. However, the participant was allowed to skip one day if the child was sick or the day was an atypical day (e.g., holiday). The mother was given an accelerometer use instruction sheet, a log sheet, a monitor set, and a survey sheet. The log sheet was used to record monitor put-on and removal time and the reason for the put-on/removal, as well as wake-up time, nap time, and bedtime. The monitor set included an ActiGraph woven nylon wrist band with an attached accelerometer, and an ActiGraph waist elastic belt with an attached accelerometer. The survey sheet included questions to assess the acceptability of wearing the device: i.e., “overall, how easy was it to have your child wear the hip monitor?” The same question was asked about the wrist monitor. The response options were: very easy, somewhat easy, somewhat challenging, and very challenging. In addition, the survey included open-ended questions to allow participants to describe the challenges and difficulties they experienced. Mothers returned the completed study package to the research site via drop-off or mail.

### 2.3. Accelerometer Data Processing

Accelerometer data were downloaded and processed to convert to 15-s epoch files using ActiLife software version 6.4 (ActiGraph Inc, Pensacola, FL, USA). According to the log sheet records, the start time and end time of monitor wear for the day, as well as intermittent non-wear time (e.g., nap time) were manually entered to identify the eligible wear time data. We additionally applied a non-wear time algorithm of consecutive zero counts for ≥20 min [7,11,16], and found that no additional data were excluded from the wear time. We extracted the following PA metrics, in hourly and daily aggregates: Vertical axis counts, vector magnitude counts, time spent in moderate- and vigorous-intensity PA (MVPA), and time spent in light-intensity PA (LPA). Hip data-based MVPA was defined as ≥420 vertical counts/15 s. Hip data-based LPA was defined as 49–419 vertical counts/15 s [24].Wrist data-based LPA and MVPA were not calculated because there was no available algorithm to define them.

### 2.4. Statistical Analysis

To evaluate acceptability of wearing the monitors, the acceptability survey question responses were dichotomized: “Very easy” and “somewhat easy” were grouped as “easy,” and “somewhat challenging” and “very challenging” were grouped as “challenging.” For the analysis of daily PA measures, any days that had ≥6 h of valid accelerometer data [25] were included. For the analysis of hourly PA measures between 8:00 AM and 7:00 PM, any hours with ≥50 min of valid accelerometer data were included. Multiple days (3 or 4 days) of PA metrics were averaged per person. Descriptive analyses for the PA metrics were conducted to calculate median, interquartile range, mean, standard deviation, and 95% confidence interval. The analyses were repeated separately for one-year olds and two-year olds, as well as separately for boys and girls.

Because the level of feasibility to acquire sufficient accelerometer data is partially determined by the requirement for valid accelerometer data (e.g., valid wear time per day required or valid wear days required), we explored variations in PA levels over time (day-by-day and hour-by-hour variations) that can inform the data requirement. To evaluate day-by-day variation of PA levels, intraclass correlation coefficients (ICC) of the daily PA metrics were calculated using the SAS MIXED procedure (two-way random effects model; mean of *k* rater; consistency definition). To compare PA levels between weekdays and weekend days, paired t-tests were conducted. Average hourly MVPA and LPA levels throughout the day were also calculated. All statistical analyses were conducted using SAS version 9.4 (SAS, Cary, NC, USA).

## 3. Results

Based on mothers’ reports of child race/ethnicity, 16 participants were non-Hispanic white, four were Asian, one was Hispanic, and one was mixed race/ethnicity. The average wake-up time was 7:08 AM (range of 5:15 AM to 8:21 AM), and average bedtime was 8:08 PM (range of 6:30 PM to 9:30 PM). The average monitor put-on time was 7:35 AM, and average monitor removal time was 7:24 PM. The difference between monitor removal time and bedtime, which was on average 44 min, was mainly due to bathing or clothing changes that occurred right before bedtime. Of the 22 toddlers, only two were reported not taking a nap during the day. Of the 20 toddlers who took a nap, average nap duration was 138 min (standard deviation (SD) of 29 min), occurring mostly between 12:00 PM and 3:00 PM.

As presented in Table 1, only one toddler refused to wear a waist belt from day 1, while five toddlers refused to wear a wrist band from day 1. One additional toddler refused to wear a wrist band from day 2. Two participants failed to comply with the four-day accelerometer protocol. One of the two failed because the mother forgot to put the monitors back on after a clothing change; and the other was reported to be sick for several days. Overall, 19 mothers (86%) reported that their child wore a waist belt for four full days, while 14 (64%) reported that their child wore a wrist band for four full days. Accelerometer data analysis revealed that 19 participants (86%) had at least three days of hip data, while 14 (64%) had at least three days of wrist data (Fisher’s Exact test *p* = 0.16). There was no significant difference in the proportion of those having ≥3 days of valid hip data between boys (*n* = 8; 80%) and girls (*n* = 11; 92%; Fisher’s Exact test *p* = 0.57). Analyzing the valid data, the median daily wear time was 499 min for hip-worn monitors and 479 min for wrist-worn monitors. Two-year olds wore the monitors longer than did one-year olds. The median wear time was similar between boys and girls (513 vs. 499 min/day).

The acceptability survey data analysis revealed that 18 mothers (82%) responded that the 4-day hip wear protocol was easy, while only 13 (59%) responded that the 4-day wrist wear protocol was easy (Fisher’s Exact test *p* = 0.19). Two mothers had expressed a concern regarding the ease of monitor wear in a childcare center setting before the monitor wear occurred. However, after the monitor wear occurred, the mothers reported that their children did not have issues wearing the monitors at the childcare center. One mother reported that her child complained about discomfort (“pain”) from wearing the wrist band. Two mothers reported that they felt that the monitor was too “big”/”bulky” for her one-year old child to wear on the wrist.

Table 2 presents the means of the daily PA measures. Based on the hip data, average daily LPA was 161 min (SD = 26 min), and average daily MVPA was 47 min (SD = 15 min). Compared to girls, boys tended to record higher LPA (171 ± 21 vs. 154 ± 25 min/day; *p* = 0.14) and MVPA (52 ± 13 vs. 44 ± 14 min/day; *p* = 0.23). One-year olds tended to have higher MVPA than two-year olds, but the difference was statistically insignificant (50 ± 15 vs. 44 ± 18 min/day; *p* = 0.49). LPA or MVPA was not significantly different between weekdays and weekend days (LPA = 163 ± 41 vs. 161 ± 42 min/day (*p* = 0.80); MVPA = 49 ± 21 vs. 46 ± 22 min/day (*p* = 0.65)). The ICC of repeatedly measured MVPA for three or four days was 0.46. Interestingly, one-year olds recorded slightly higher hip vector magnitude counts, but significantly lower wrist vector magnitude counts, compared to two-year olds.

**Figure 1:**
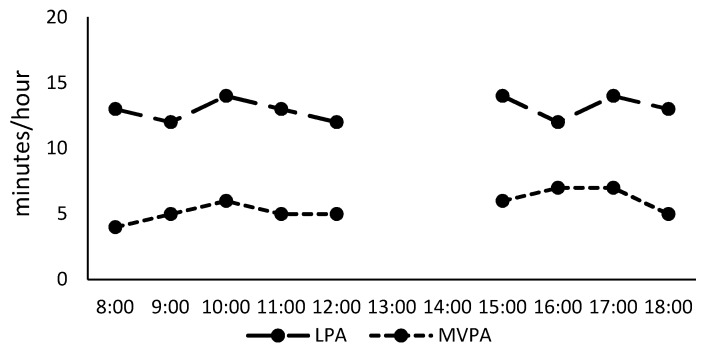
Means of hourly light-intensity physical activity (LPA) and moderate- and vigorous-intensity physical activity (MVPA) levels between 8:00 AM and 7:00 PM among toddlers.

Figure 1 illustrates the means of hourly LPA and MVPA minutes over a day. Because 17 of 19 participants (89%) did not have valid hourly data between 1:00 PM and 3:00 PM, mostly due to taking a nap during those hours, we did not present hourly data between 1:00 PM and 3:00 PM. LPA and MVPA levels were stable throughout the day while toddlers were awake.

## 4. Discussion

This study aimed to compare the feasibility of hip vs. wrist accelerometers in measuring free-living PA among toddlers and to describe accelerometer-derived PA metrics. The findings from this study support that hip placement is more feasible than wrist placement among toddlers. The median accelerometer wear time was approximately 8 h per day for one-year olds, and approximately 9 h per day for two-year olds.

Although the available research is limited, studies [6,10] have reported a relatively low compliance rate for ActiGraph accelerometer data collection protocols among toddlers. In an epidemiologic study by Costa et al. [6] that requested toddler participants and their parents to wear an ActiGraph accelerometer on the hip for 8 days, only 75% of toddlers had enough valid data (defined as at least three days of waking hour data). This was in contrast to 91% of their mothers, who had enough valid data. A study by Hager et al. [10], which requested toddlers to wear an Actical accelerometer on the ankle for at least seven consecutive days without removal (24 h/7 days), reported 69% compliance (defined as at least three days of 24-h data). This current study suggests that hip placement is more feasible than wrist placement among toddlers. Given that hip data reflect physical activity behaviors of interest more accurately than wrist data among toddlers [26] and older children [24], the current evidence supports a hip placement method over a wrist placement method among toddlers for higher compliance and accuracy.

Several studies [6,7,8,9,10,11,12,13,14,15,16,17,18] have investigated PA levels in free-living settings among toddlers. These studies have reported a wide range of daily PA levels using different accelerometer count cut-points to define MVPA (Table 3). Of them, the Parents’ Role in Establishing Healthy Physical Activity and Sedentary Behavior Habits (PREPS) study [18] reported 58 min/day of MVPA among children aged approximately 19 months, using the same definition of MVPA as the current study. The current study sample engaged in 11 min less MVPA on average than the PREPS sample (47 vs. 58 min/day).

**Figure 2:**
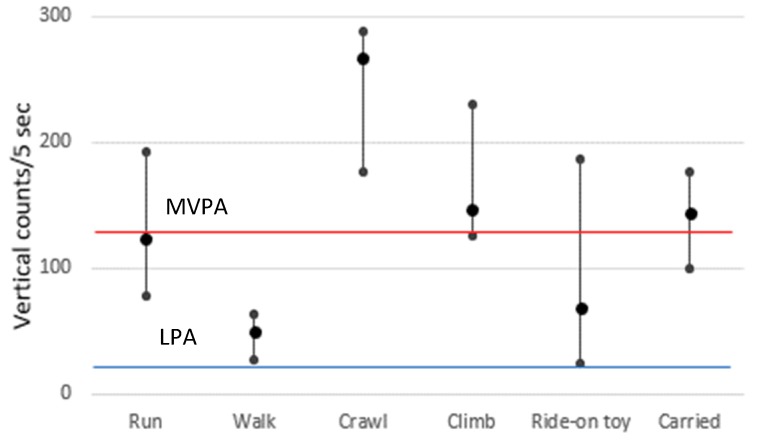
Median and Interquartile range of hip vertical accelerometer counts per 5 s (reproduction of the data published in Kwon et al.) [26]. * Note: The blue line indicates the lower threshold for LPA; the red line indicates the lower threshold for MVPA.

This wide variation in MVPA across studies is not only due to variation in the study populations, but also due to the use of different definitions for MVPA. It is also important to understand which activities are captured as MVPA, particularly given that toddlers engage in unique activities, such as being “carried” by an adult or riding in a stroller, that are observed less frequently, or not at all, among older children. In a separate pilot study, our research group [26] demonstrated that all walking activities of toddlers would be classified as LPA, not MVPA (Figure 2). This is presumably due to a small vertical acceleration associated with their short stature [26]. In contrast, more than half of “carried” activities would be misclassified as MVPA [26], because high vertical counts are recorded while toddlers are “carried,” similar to the counts recorded while running. This observation presumably reflects the ambulatory movements conducted by the carrying adult [26]. Alternatively, LPA could be used as a PA measure for toddlers, as LPA appears to be more inclusive (since it includes walking), but still excludes typical sedentary behaviors, such as sitting or riding in a stroller (a median of 0 vertical counts [26]; not shown in Figure 2). However, “carried” activities would still be misclassified. Our research group [26] also explored a machine learning activity recognition algorithm to differentiate being “carried” activities from ambulation. This methodology is still in the very early stages of its validation and requires further investigation.

The current study found no difference in PA levels between weekdays and weekend days, which is consistent with a previous study among children aged 1 and 3 years [20]. Further, the current study found that day-by-day PA levels for three or four days were highly correlated. The data requirement of three valid days could be acceptable to achieve a reliable assessment of habitual PA among toddlers, as a previous study [27] indicated that 3-day PA data with daily monitor wear time ≥9 h would achieve acceptable reliability (70%) in preschoolers aged 3 to 5 years. However, the current study was unable to test the reliability by the number of valid days required, because the maximum wear day period was only four days. Further investigation is needed to assess the volume of accelerometer data required for acceptable reliability among toddlers. The finding of a small variation in PA levels throughout the day, which is similar to the findings of a previous study [7], suggests a great potential to develop a reliable imputation algorithm for missing accelerometer data during intermittent non-wear time among toddlers.

Several limitations should be acknowledged. First, this study had a small sample size. A statistically insignificant difference in some group comparisons (e.g., by sex) could be partly due to a lack of power. Second, this study used a convenience sample, which was not racially/ethnically diverse. Thus, the study results from this convenience sample may not be generalizable to other populations. Third, because only four-day monitor wear was tested, caution is required to directly compare the data from a seven-day monitor wear protocol. Lastly, this study relied on caregivers to log information, which could be inaccurate. Nonetheless, this study is one of very few studies to compare the feasibility of hip-worn vs. wrist-worn ActiGraph accelerometer use among toddlers. This study also suggests guidance for future PA assessment studies among toddlers.

In conclusion, this study suggests that hip-worn accelerometer placement is more feasible than wrist-worn accelerometer placement among toddlers.

## Figures and Tables

**Table 1:** Compliance with the accelerometer monitoring protocol among toddlers.

Item	Hip			Wrist		
	Total	1 Year Old	2 Years Old	Total	1 Year Old	2 Years Old
Participants, *n*	22	11	11	22	11	11
Toddler refusals to wear monitors, *n*	1	0	1	6	4	2
≥3 days of wear, *n*	19	10	9	14	6	8
Daily wear minutes, median (min, max)	499	491	545	479	440	525
	(403, 595)	(403, 594)	(416, 595)	(345, 634)	(345, 487)	(417, 634)

**Table 2:** Means and 95% confidence intervals of daily physical activity measures among toddlers.

Item	Hip			Wrist		
	Total	1 Year Old	2 Years Old	Total	1 Year Old	2 Years Old
Vertical counts, x 10^3^ counts/day	254	257	252	1069	938	1163
	(226, 283)	(213, 300)	(200, 304)	(875, 1264)	(467, 1409)	(954, 1372)
Vector magnitude, x 10^3^ counts/day	590	599	581	1814	1559	1996
	(535, 645)	(512, 685)	(486, 675)	(1492, 2135)	(835, 2282)	(1638, 2354)
LPA, mins/day	161 (147, 176)	156 (133, 178)	168 (144, 192)	NA	NA	NA
MVPA, mins/day	47 (39, 56)	50 (38, 62)	44 (29, 60)	NA	NA	NA

NA, not applicable as the definitions of LPA and MVPA are unavailable for wrist data.

**Table 3:** Moderate- and vigorous-intensity physical activity (MVPA) levels among toddlers reported in previous and current studies.

Study	Sample	MVPA Definition	MVPA, Mins/Day
InFANT study [7]	295 children aged 18–19 months	>1672 Hip-worn ActiGraph counts/min	48
TARGet Kids! Study [8]	28 children aged < 18 months	≥2860 hip-worn Actical counts/min	4
TARGet Kids! Study [8]	45 children aged 18–59 months old	≥2860 hip-worn Actical counts/min	29
Hager et al. [10]	191 children aged 12–36 months	≥2201 ankle-worn Actical counts/min	54
Alberta childcare study [11]	114 children aged 19–60 months	≥287.5 hip-worn Actical counts/15 s	4
Generation R study [14]	347 children aged 2 years	≥615 hip-worn ActiGraph accelerometer counts/15 s	25
Early STOPP study [15]	123 children aged 2 years	>440 wrist-worn ActiGraph accelerometer counts/5 s *	84
PREPS study [18]	149 children aged 19 months on average (12–23 months)	>420 Hip-worn ActiGraph counts/15 s	58
The current study	19 children aged 13–35 months	≥420 Hip-worn ActiGraph counts/15 s	47

* Definition for high-intensity activity.

## References

[1] Physical Activity Guidelies for Americans Committee (2018). 2018 Physical Activity Guidelines Advisory Committee Scientific Report.

[2] National Physical Activity Plan Alliance (2018). The 2018 United States Report Card on Physical. Activity for Children and Youth.

[3] Kwon S., Janz K., Letuchy E., Trudy B., Steven L. (2015). Developmental trajectories of physical activity, sports, and television viewing during childhood to young adulthood. Pediatrics.

[4] Carson V., Lee E.Y., Hesketh K.D. (2019). Physical activity and sedentary behavior across three time-points and associations with social skills in early childhood. BMC Public Health.

[5] Meredith-Jones K., Haszard J., Moir C. (2018). Physical activity and inactivity trajectories associated with body composition in pre-schoolers. Int. J. Obes..

[6] Costa S., Barber S.E., Cameron N., Clemes S.A. (2015). The objective measurement of physical activity and sedentary behaviour in 2–3 year olds and their parents: A cross-sectional feasibility study in the bi-ethnic Born in Bradford cohort. BMC Public Health.

[7] Hnatiuk J., Ridgers N.D., Salmon J., Campbell K., McCallum Z., Hesketh K. (2012). Physical activity levels and patterns of 19-month-old children. Med. Sci. Sports Exerc..

[8] Borkhoff C.M., Heale L.D., Anderson L.N. (2015). Objectively measured physical activity of young Canadian children using accelerometry. Appl. Physiol. Nutr. Metab..

[9] Lee E.Y., Hesketh K.D., Rhodes R.E., Rinaldi C.M., Spence J.C., Carson V. (2018). Role of parental and environmental characteristics in toddlers’ physical activity and screen time: Bayesian analysis of structural equation models. Int. J. Behav. Nutr. Phys. Act..

[10] Hager E.R., Gormley C.E., Latta L.W., Treuth M.S., Caulfield L.E., Black M.M. (2016). Toddler physical activity study: Laboratory and community studies to evaluate accelerometer validity and correlates. BMC Public Health..

[11] Kuzik N., Clark D., Ogden N., Harber V., Carson V. (2015). Physical activity and sedentary behaviour of toddlers and preschoolers in child care centres in Alberta, Canada. Can. J. Public Health.

[12] Raza H., Zhou S.M., Todd C. (2019). Predictors of objectively measured physical activity in 12-month-old infants: A study of linked birth cohort data with electronic health records. Pediatr. Obes..

[13] Armstrong B., Covington L.B., Hager E.R., Black M.M. (2019). Objective sleep and physical activity using 24-h ankle-worn accelerometry among toddlers from low-income families. Sleep Health.

[14] Wijtzes A.I., Kooijman M.N., Kiefte-de Jong J.C. (2013). Correlates of physical activity in 2-year-old toddlers: The generation R study. J. Pediatr..

[15] Johansson E., Hagströmer M., Svensson V. (2015). Objectively measured physical activity in two-year-old children-levels, patterns and correlates. Int. J. Behav. Nutr. Phys. Act..

[16] Lee E.Y., Hesketh K.D., Hunter S. (2017). Meeting new Canadian 24-Hour Movement Guidelines for the Early Years and associations with adiposity among toddlers living in Edmonton, Canada. BMC Public Health.

[17] Hnatiuk J., Salmon J., Campbell K.J., Ridgers N.D., Hesketh K.D. (2013). Early childhood predictors of toddlers’ physical activity: Longitudinal findings from the Melbourne InFANT Program. Int. J. Behav. Nutr. Phys. Act..

[18] Hunter S., Rosu A., Hesketh K.D. (2019). Objectively Measured Environmental Correlates of Toddlers’ Physical Activity and Sedentary Behavior. Pediatr. Exerc. Sci..

[19] Cain K.L., Sallis J.F., Conway T.L., Van Dyck D.L. (2013). Using accelerometers in youth physical activity studies: A review of methods. J. Phys. Act. Health.

[20] Van Cauwenberghe E., Gubbels J., De Bourdeaudhuij I., Cardon G. (2011). Feasibility and validity of accelerometer measurements to assess physical activity in toddlers. Int. J. Behav. Nutr. Phys. Act..

[21] Fairclough S.J., Noonan R., Rowlands A.V., Van Hees V., Knowles Z., Boddy L.M. (2016). Wear Compliance and Activity in Children Wearing Wrist- and Hip-Mounted Accelerometers. Med. Sci. Sports Exerc..

[22] Trost S.G., Zheng Y., Wong W.K. (2014). Machine learning for activity recognition: Hip versus wrist data. Physiol. Meas..

[23] Ellis K., Kerr J., Godbole S., Staudenmayer J., Lanckriet G. (2016). Hip and Wrist Accelerometer Algorithms for Free-Living Behavior Classification. Med. Sci. Sports Exerc..

[24] Bisson M., Tremblay F., Pronovost E., Julien A.S., Marc I. (2019). Accelerometry to measure physical activity in toddlers: Determination of wear time requirements for a reliable estimate of physical activity. J. Sports Sci..

[25] Trost S.G., Fees B.S., Haar S.J., Murray A.D., Crowe L.K. (2012). Identification and validity of accelerometer cut-points for toddlers. Obesity.

[26] Kwon S., Zavos P., Nickele K., Sugianto A., Albert M.A. (2019). Hip and wrist-worn accelerometer data analysis for toddler activities. Int. J. Environ. Res. Public Health.

[27] Hinkley T., O’Connell E., Okely A.D., Crawford D., Hesketh K., Salmon J. (2012). Assessing volume of accelerometry data for reliability in preschool children. Med. Sci. Sports Exerc..

